# Correction: Prevalence of diarrheal diseases and associated factors among under five children in Africa: A meta-analysis

**DOI:** 10.1371/journal.pone.0344212

**Published:** 2026-03-02

**Authors:** Eshetu Abera Worede, Asmamaw Malede, Hailemariam Feleke, Geziew Abere, Eyayaw Addissu Demeke, Jember Azanaw

The fourth author’s name is spelled incorrectly. The correct name is: Giziew Abere. The correct citation is: Worede EA, Malede A, Feleke H, Abere G, Demeke EA, Azanaw J (2025) Prevalence of diarrheal diseases and associated factors among under five children in Africa: A meta-analysis. PLoS One 20(7): e0326501. https://doi.org/10.1371/journal.pone.0326501
