# Supplementary material for: Prevalence of diarrheal diseases and associated factors among under five children in Africa: A meta-analysis
Source: PLoS One. 2025 Jul 3;20(7):e0326501. doi: 10.1371/journal.pone.0326501 (PMC12225826; doi:10.1371/journal.pone.0326501)
Supplement: S3 File — (PDF) [file pone.0326501.s004.pdf]

Newcastle Ottawa Scale Quality Assessment checklist for quality Assessment of included studies

Author, year of publication and Reference	Q1	Q2	Q3	Q4	Q5	Q6	Q7	Q8	Q9	Total score (9)
Tadesse et al. (2022)	Y	Y	Y	Y	Y	Y	Y	Y	Y	9
Getahunet al. (2021)	Y	Y	Y	Y	Y	Y	Y	Y	Y	9
Mohammed et al. (2013)	Y	Y	Y	Y	Y	Y	Y	Y	Y	9
Natnael et al. (2021)	Y	Y	NA	Y	Y	Y	Y	Y	Y	8
Girmay et al. (2023)	Y	Y	Y	Y	Y	Y	Y	Y	Y	9
Bitew et al. (2023)	Y	Y	Y	Y	Y	Y	Y	Y	Y	8
Godana et al. (2013)	Y	Y	Y	Y	Y	Y	Y	Y	Y	9
Nantege et al. (2022)	NA	Y	NA	Y	Y	Y	Y	Y	Y	7
Asgedom et al. (2023)	NA	Y	Y	Y	Y	Y	Y	Y	Y	8
Thiam et al. (2017)	Y	Y	Y	Y	Y	Y	Y	Y	Y	9
Omona et al. (2020)	Y	Y	Y	Y	Y	Y	Y	Y	Y	9
Mulatu et al. (2022)	Y	Y	Y	Y	Y	Y	Y	Y	Y	9
Birhan et al. (2023)	Y	Y	Y	Y	Y	Y	Y	Y	Y	9
Workie et al. (2019)	Y	Y	Y	Y	Y	Y	Y	Y	Y	9
Gizaw et al. (2017)	Y	Y	NA	Y	Y	Y	Y	Y	Y	8
Melese et al. (2023)	Y	Y	Y	Y	Y	Y	Y	Y	Y	8
Anteneh et al. (2017)	Y	Y	Y	Y	Y	Y	Y	Y	Y	9
Gebru et al. (2014)	Y	Y	Y	Y	Y	Y	Y	Y	Y	8

Mihrete et al. (2014)	Y	Y	Y	Y	Y	Y	Y	Y	Y	9
McClelland et al. (2022)	NA	Y	NA	Y	Y	Y	Y	Y	Y	7
Woldu et al. (2016)	NA	Y	Y	Y	Y	Y	Y	Y	Y	8
Hashi et al. (2016)	Y	Y	Y	Y	Y	Y	Y	Y	Y	9
Alemayehu et al. (2020)	Y	Y	Y	Y	Y	Y	Y	Y	Y	9
Siziya et al. (2013)	Y	Y	Y	Y	Y	Y	Y	Y	Y	9
Wasihun et al. (2018)	Y	Y	Y	Y	Y	Y	Y	Y	Y	9
Machava et al. (2022)	Y	Y	Y	Y	Y	Y	Y	Y	Y	9
Akinyemi et al. (2019)	Y	Y	NA	Y	Y	Y	Y	Y	Y	8
Asfaha et al. (2018)	Y	Y	Y	Y	Y	Y	Y	Y	Y	8
Tambe et al. (2015)	Y	Y	Y	Y	Y	Y	Y	Y	Y	9
Mengistie et al. (2013)	Y	Y	Y	Y	Y	Y	Y	Y	Y	8
Melese et al. (2019)	Y	Y	Y	Y	Y	Y	Y	Y	Y	9
Soboksa et al. (2021)	NA	Y	NA	Y	Y	Y	Y	Y	Y	7
Kefalew et al. (2021)	Y	Y	Y	Y	Y	Y	Y	Y	Y	9
Dagnew et al. (2019)	Y	Y	Y	Y	Y	Y	Y	Y	Y	9
Nwokoro et al. (2020)	Y	Y	Y	Y	Y	Y	Y	Y	Y	9
Diouf et al. (2014)	Y	Y	NA	Y	Y	Y	Y	Y	Y	8
Colombo et al. (2023)	Y	Y	Y	Y	Y	Y	Y	Y	Y	8
Danquah et al. (2014)	Y	Y	Y	Y	Y	Y	Y	Y	Y	9
Naah et al. (2019)	Y	Y	Y	Y	Y	Y	Y	Y	Y	9
Abu et al (2018)	NA	Y	NA	Y	Y	Y	Y	Y	Y	7

Apanga et al. (2021)	NA	Y	Y	Y	Y	Y	Y	Y	Y	8
Raza et al. (2020)	Y	Y	Y	Y	Y	Y	Y	Y	Y	9
Guillaume et al. (2020)	Y	Y	Y	Y	Y	Y	Y	Y	Y	9
Fagbamigbe et al. (2017)	Y	Y	Y	Y	Y	Y	Y	Y	Y	9
Onyearugha et al. (2020)	Y	Y	Y	Y	Y	Y	Y	Y	Y	9
Yaya et al. (2018)	Y	Y	Y	Y	Y	Y	Y	Y	Y	9
Ntshangase et al. (2022)	Y	Y	NA	Y	Y	Y	Y	Y	Y	8
Birungi et al. (2016)	Y	Y	Y	Y	Y	Y	Y	Y	Y	8
Kapwata et al. (2018)	Y	Y	Y	Y	Y	Y	Y	Y	Y	9
Claudine et al. (2021)	Y	Y	Y	Y	Y	Y	Y	Y	Y	8
Bennion et al. (2021)	Y	Y	Y	Y	Y	Y	Y	Y	Y	9
Rukambile et al. (2023)	NA	Y	NA	Y	Y	Y	Y	Y	Y	7
Bah et al. (2022)	Y	Y	Y	Y	Y	Y	Y	Y	Y	9
Atari et al. (2021)	Y	Y	Y	Y	Y	Y	Y	Y	Y	9
Daffe et al. (2022)	Y	Y	Y	Y	Y	Y	Y	Y	Y	9
Moon et al. (2019)	Y	Y	Y	Y	Y	Y	Y	Y	Y	9
Awoniyi et al. (2021)	Y	Y	NA	Y	Y	Y	Y	Y	Y	8
Elmanssury et al. (2022)	Y	Y	Y	Y	Y	Y	Y	Y	Y	8
Netsereab et al. (2017)	Y	Y	Y	Y	Y	Y	Y	Y	Y	9
Jayte et al. (2023)	Y	Y	Y	Y	Y	Y	Y	Y	Y	8
Chilambwe et al. (2015)	Y	Y	Y	Y	Y	Y	Y	Y	Y	9
Oyedele et al. (2023)	NA	Y	NA	Y	Y	Y	Y	Y	Y	7

Dharod et al. (2021)	NA	Y	Y	Y	Y	Y	Y	Y	Y	8
Lanyero et al. (2021)	Y	Y	Y	Y	Y	Y	Y	Y	Y	9
Aderinwale et al. (2023)	Y	Y	Y	Y	Y	Y	Y	Y	Y	9
William et al. (2022)	Y	Y	Y	Y	Y	Y	Y	Y	Y	9

Key: Y= Yes; **NR**= Not reported, **NA**=Not appropriate

Question codes:

Q1. Was the sample frame appropriate to address the target population?

Q2. Were study participants sampled in an appropriate way?

Q3. Was the sample size adequate?

Q4. Were the study subjects and the setting described in detail?

Q5. Was the data analysis conducted with sufficient coverage of the identified sample?

Q6. Were valid methods used for the identification of the condition?

Q7. Was the condition measured in a standard, reliable way for all participants?

Q8. Was there appropriate statistical analysis?

Q9. Was the response rate adequate, and if not, was the low response rate managed appropriately?

Newcastle Ottawa Scale quality assessment checklist for the pooled prevalence of Diarrhea and associated factors in Africa among children under five.
