# Supplementary figures and images for: Prevalence of diarrheal diseases and associated factors among under five children in Africa: A meta-analysis
Source: PLoS One. 2025 Jul 3;20(7):e0326501. doi: 10.1371/journal.pone.0326501 (PMC12225826; doi:10.1371/journal.pone.0326501)

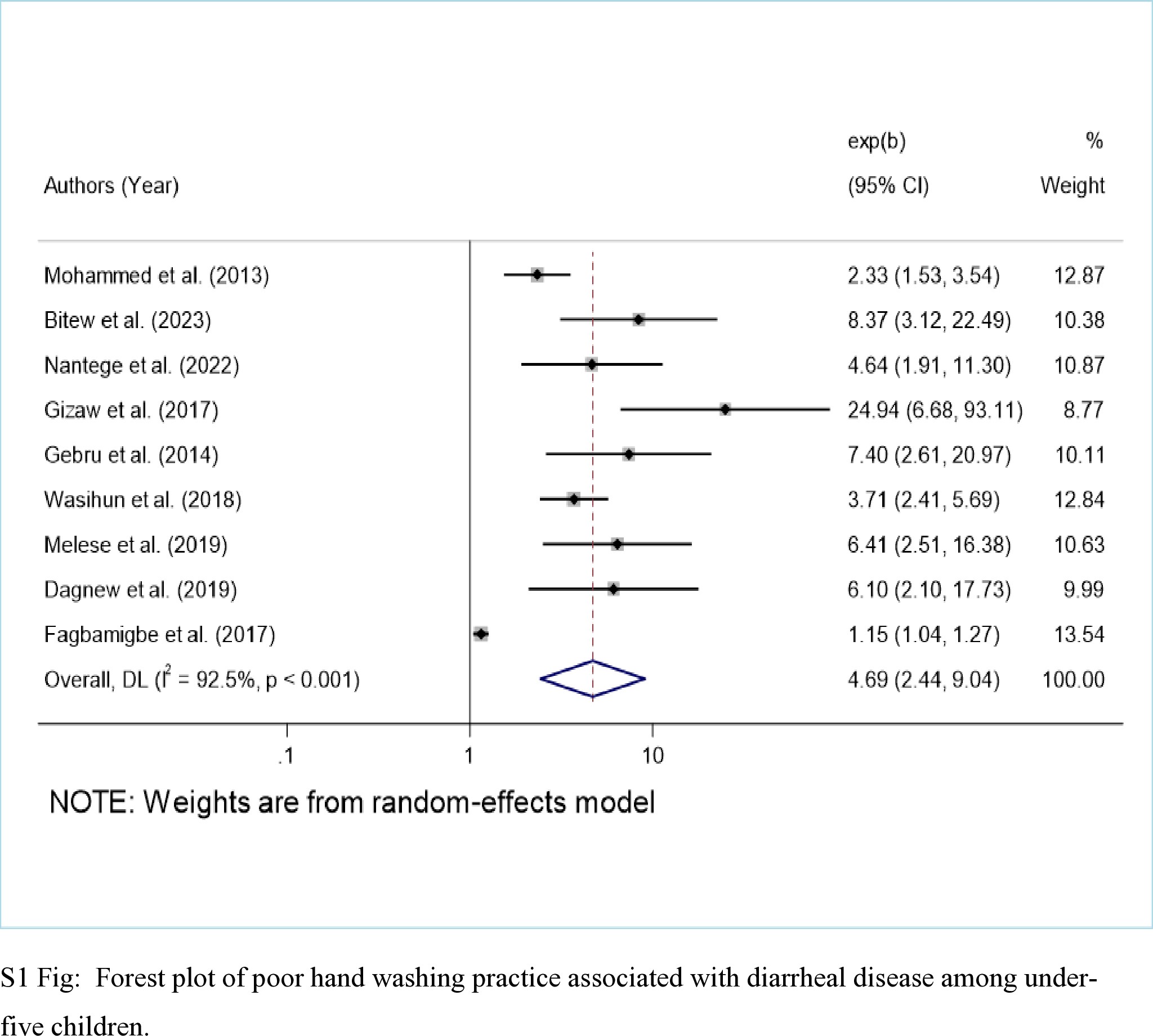

Supplement: S1 Fig — (TIF) [file pone.0326501.s005.TIF]

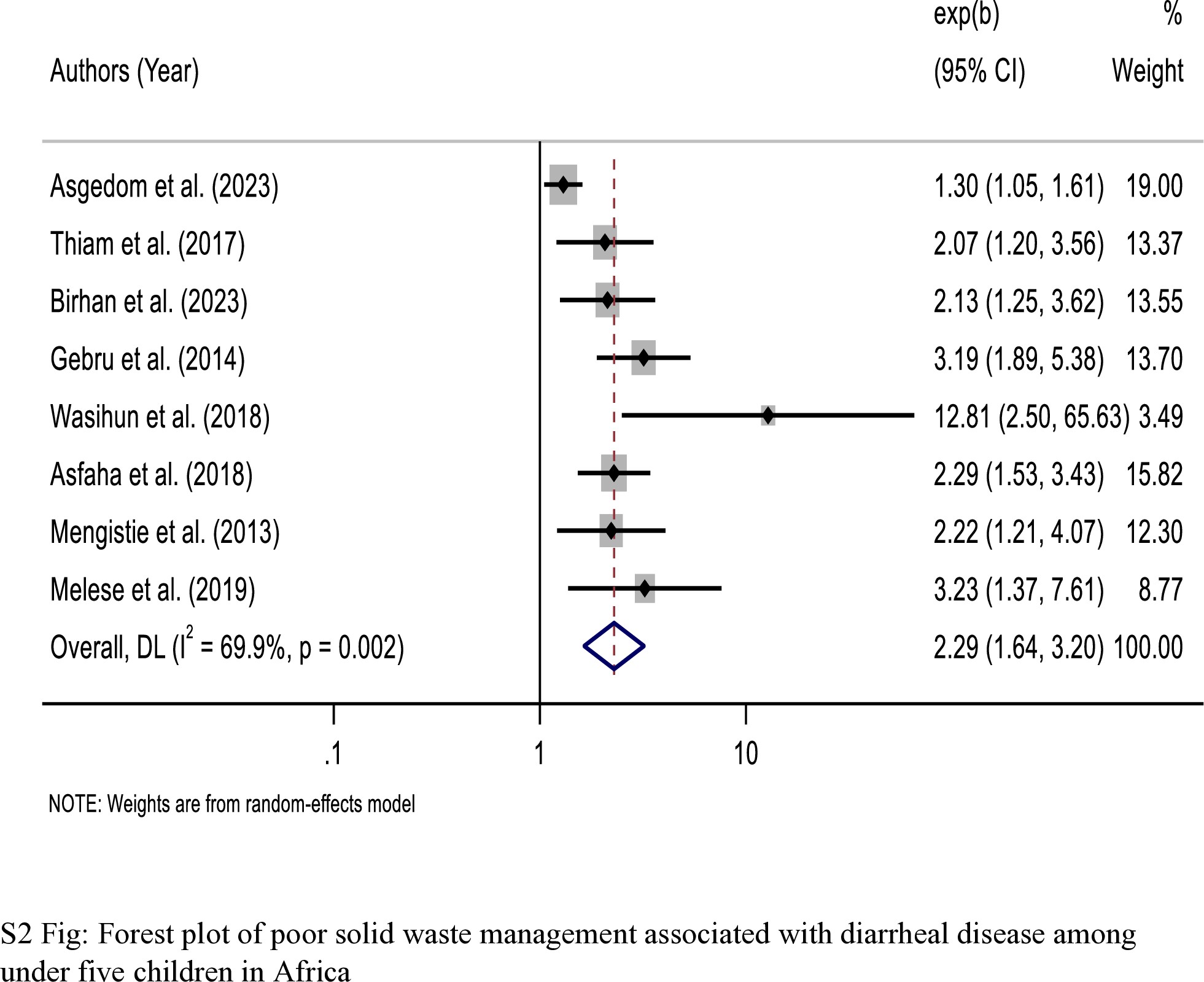

Supplement: S2 Fig — (TIF) [file pone.0326501.s006.TIF]

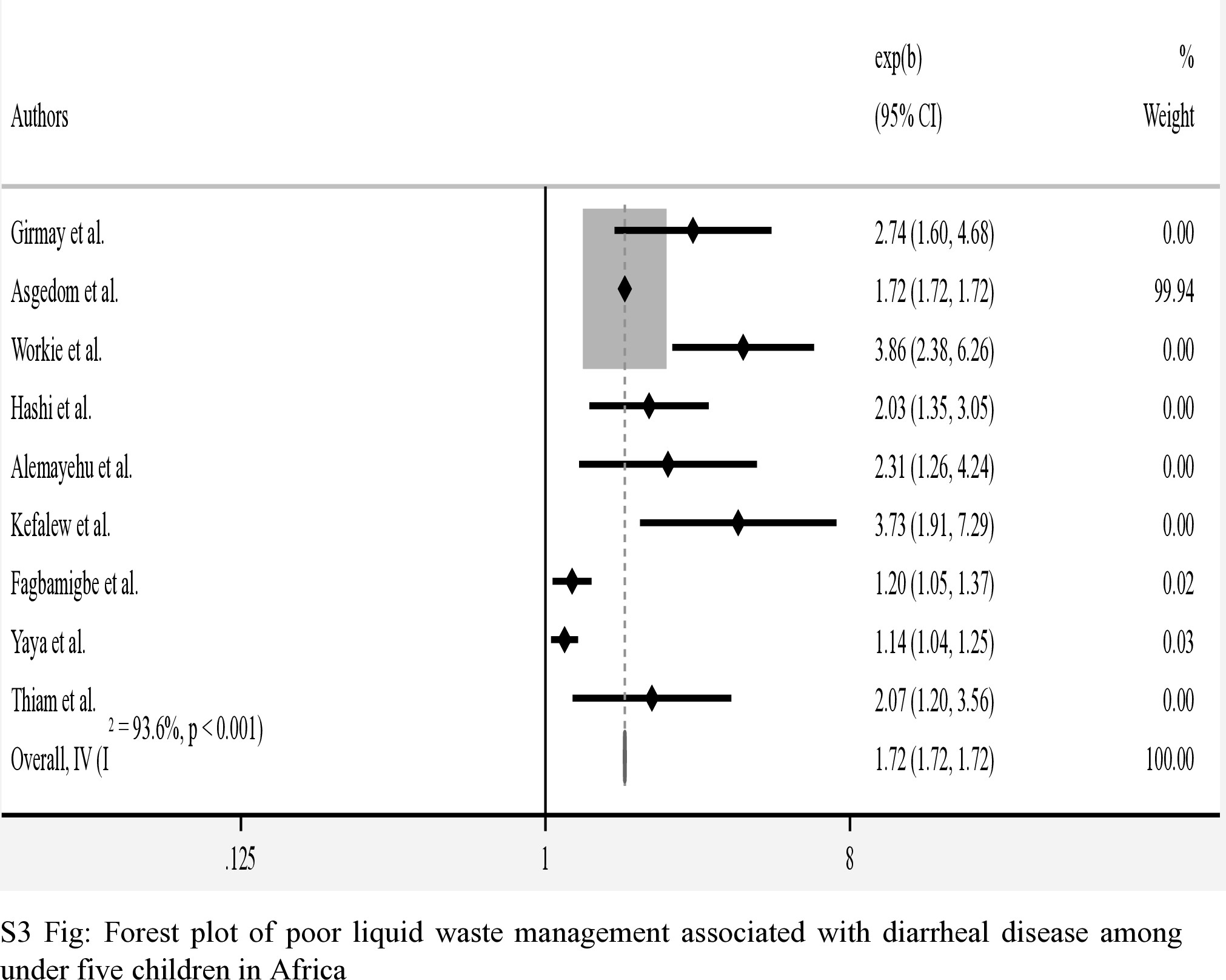

Supplement: S3 Fig — (TIF) [file pone.0326501.s007.TIF]

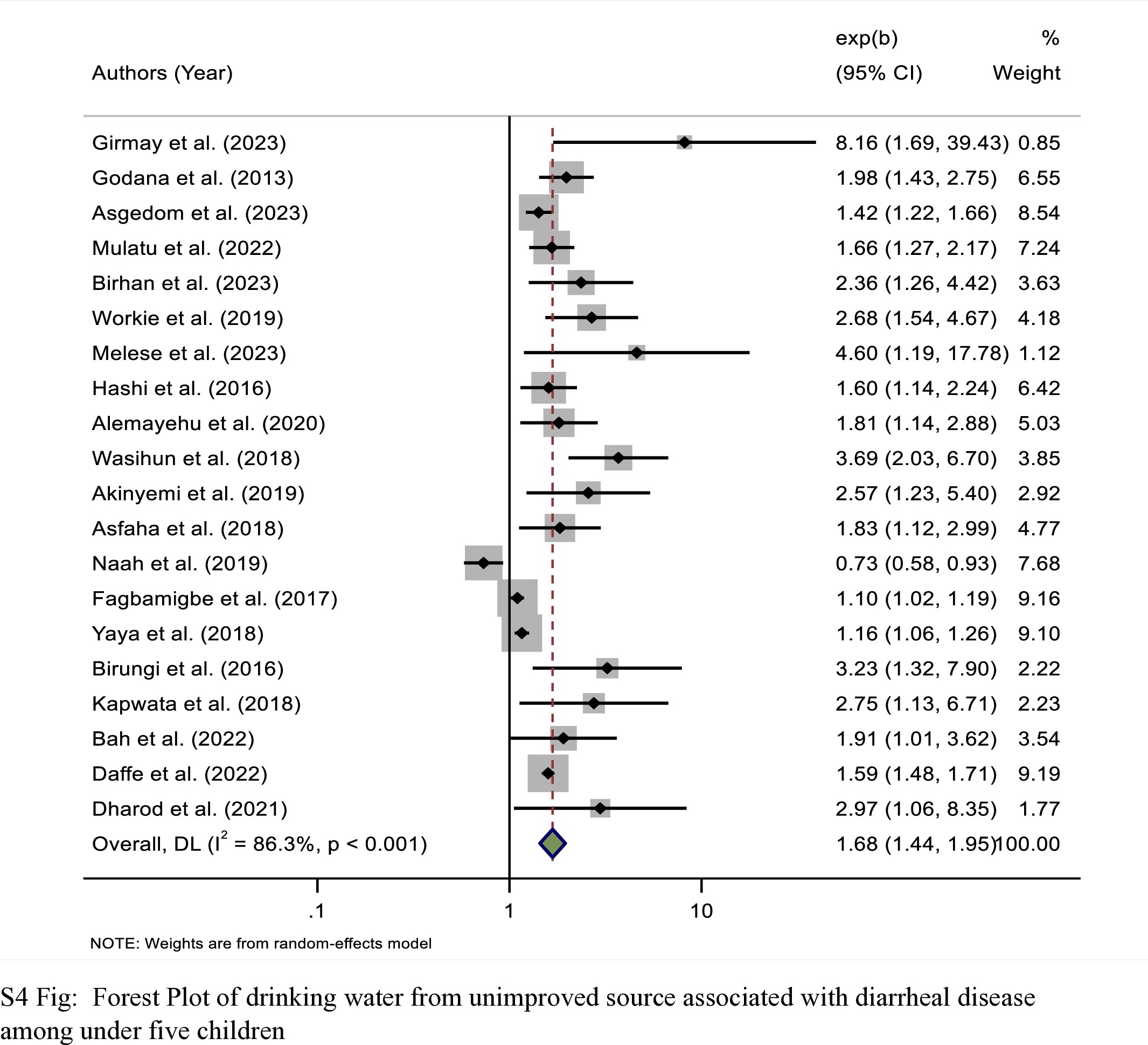

Supplement: S4 Fig — (TIF) [file pone.0326501.s008.TIF]

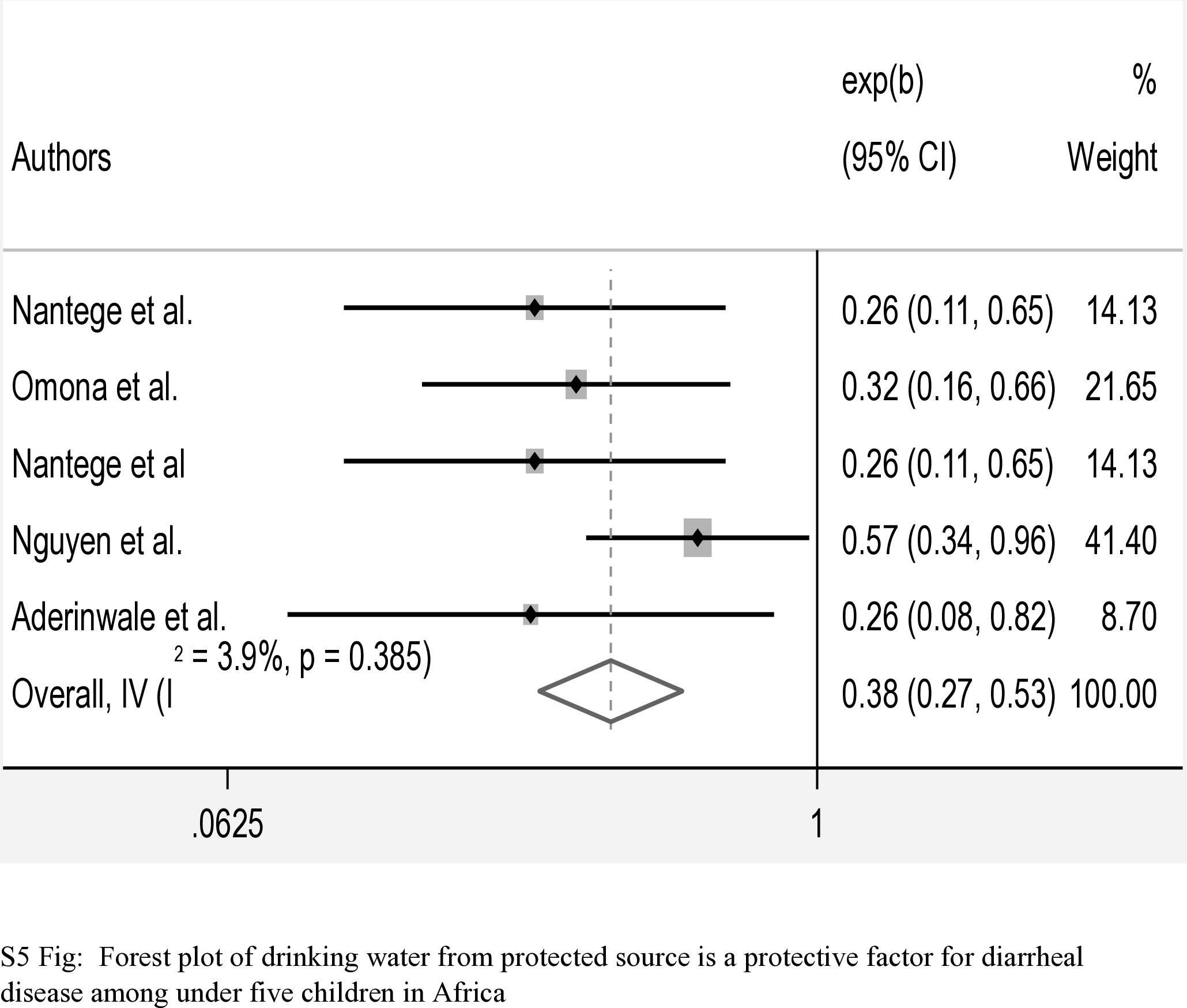

Supplement: S5 Fig — (TIF) [file pone.0326501.s009.TIF]

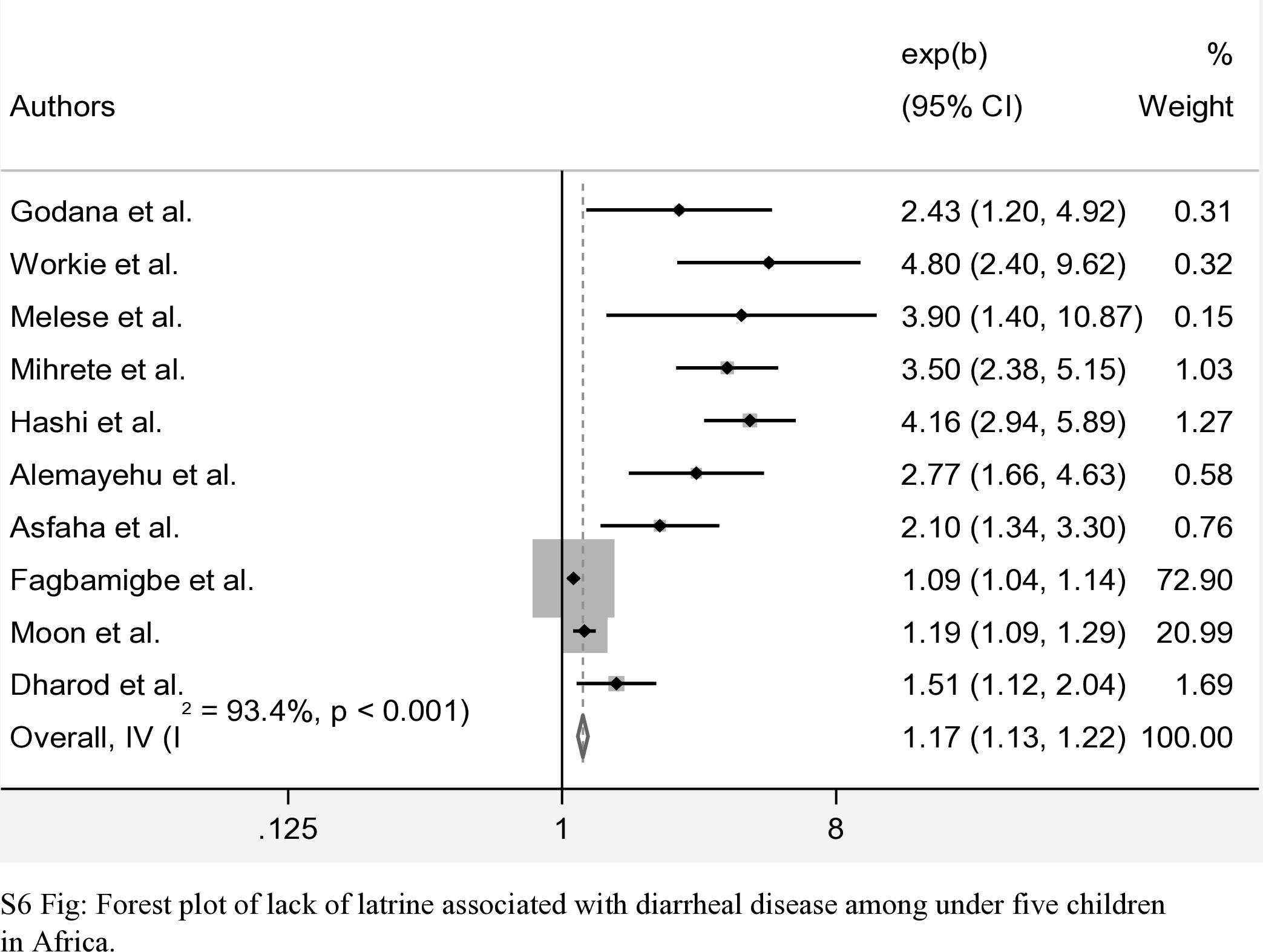

Supplement: S6 Fig — (TIF) [file pone.0326501.s010.TIF]

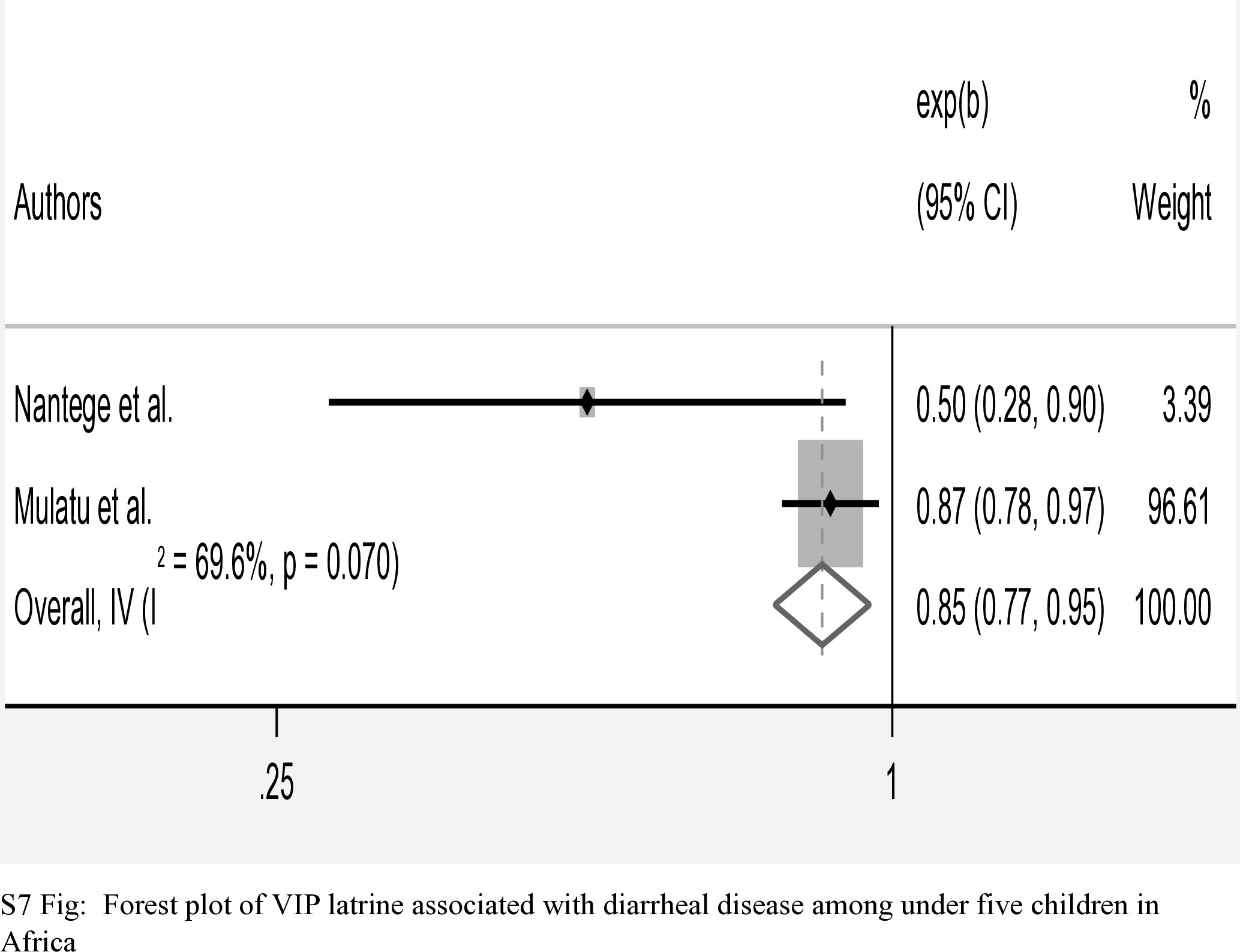

Supplement: S7 Fig — (TIF) [file pone.0326501.s011.TIF]

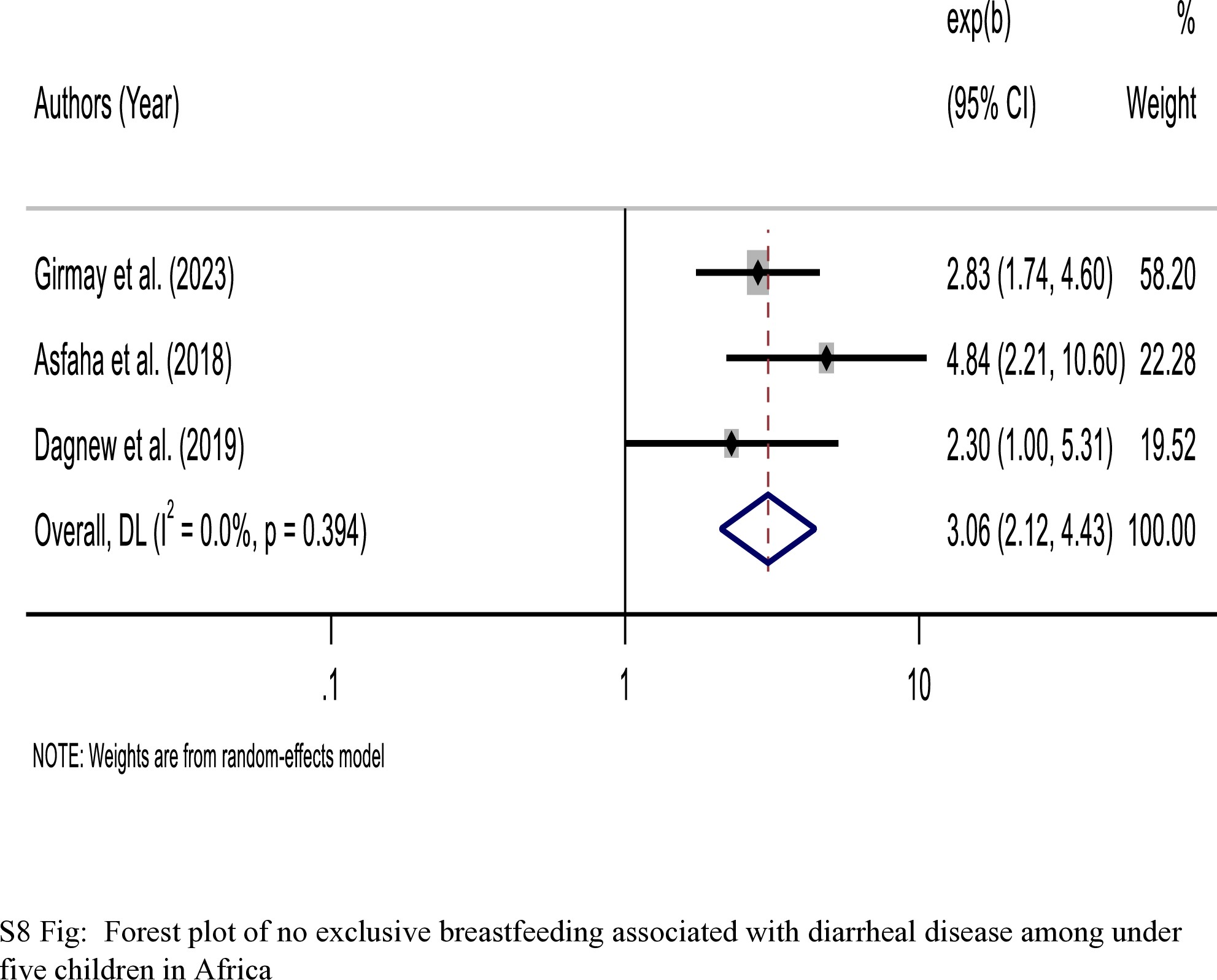

Supplement: S8 Fig — (TIF) [file pone.0326501.s012.TIF]

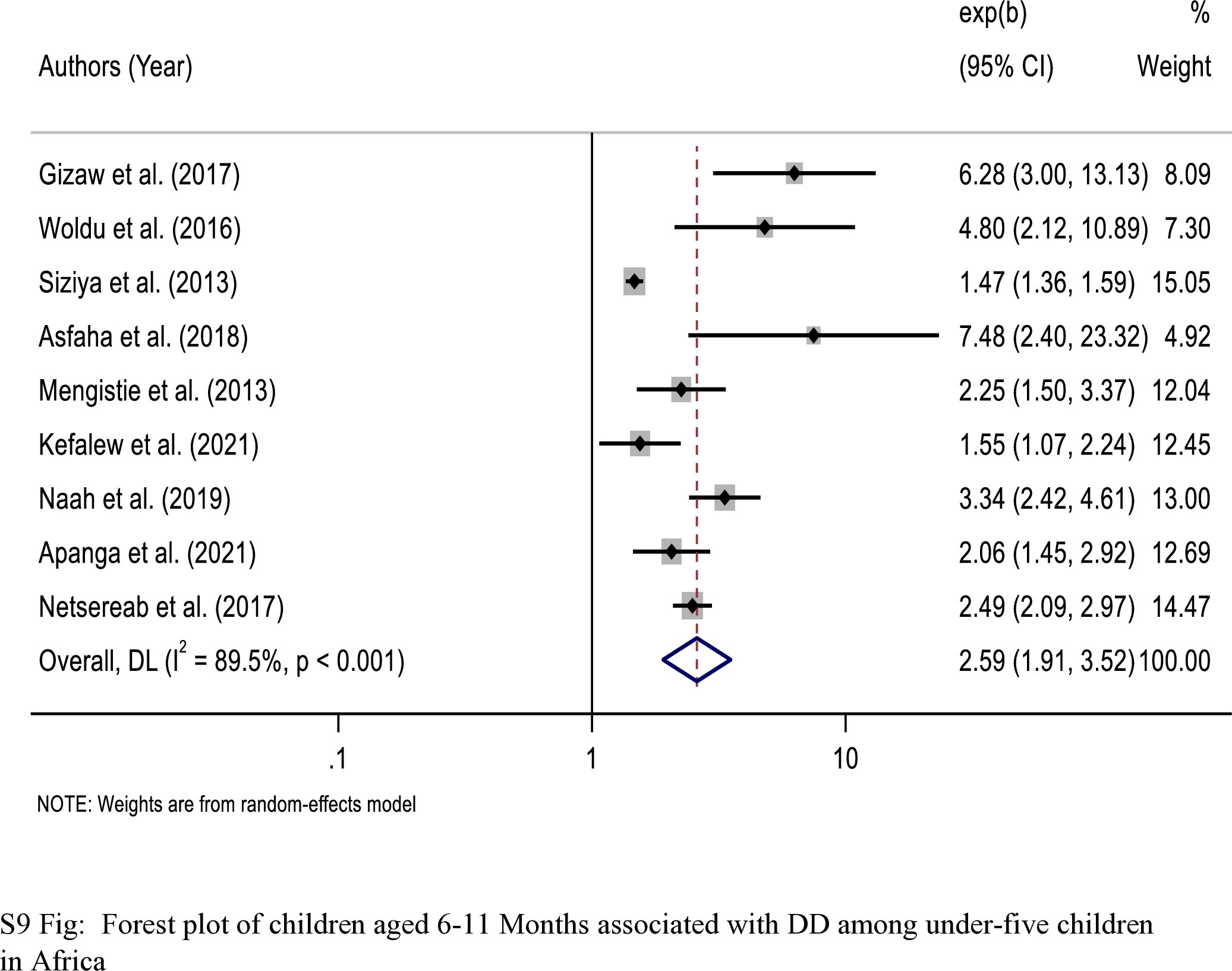

Supplement: S9 Fig — (TIF) [file pone.0326501.s013.TIF]

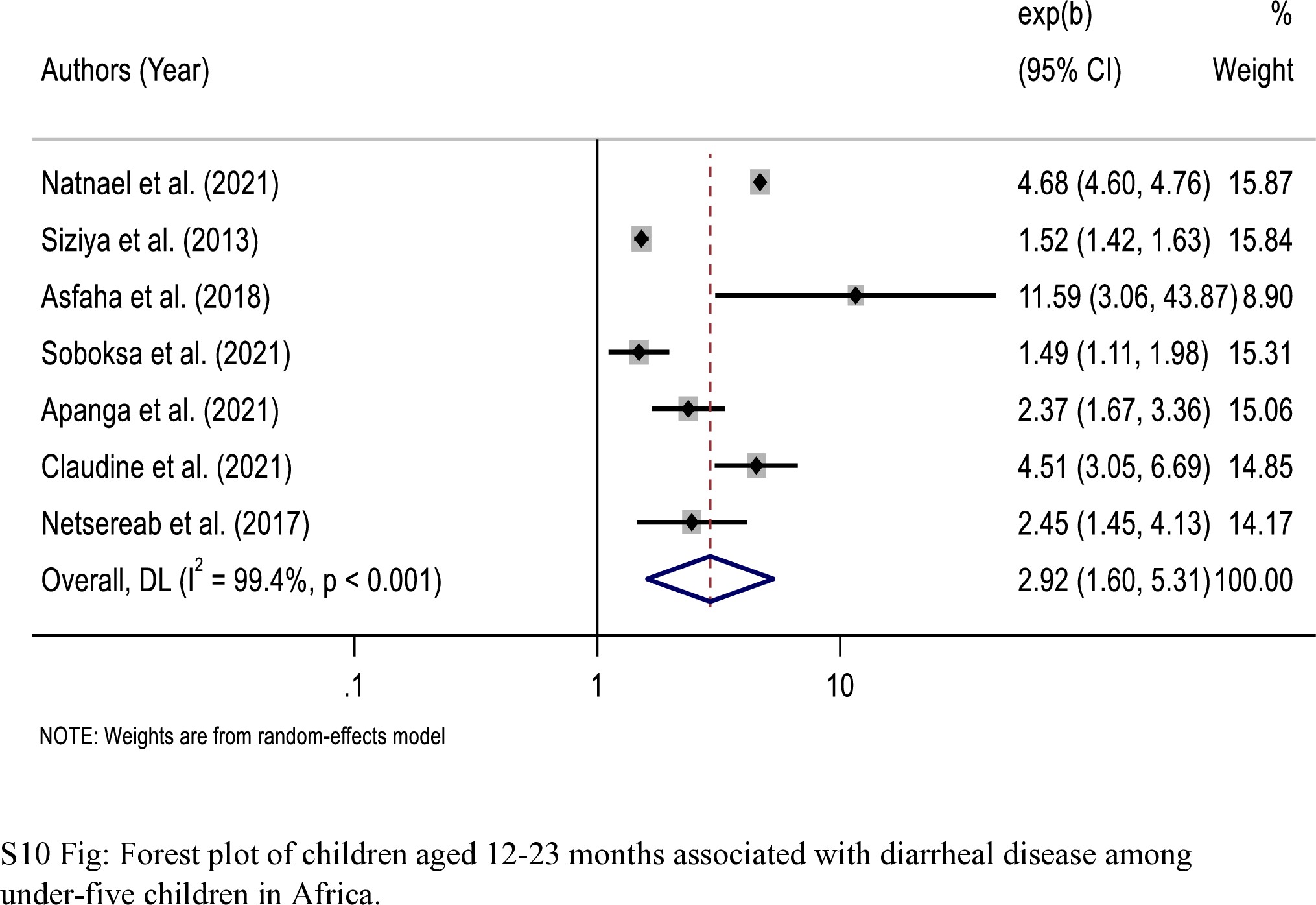

Supplement: S10 Fig — (TIF) [file pone.0326501.s014.TIF]

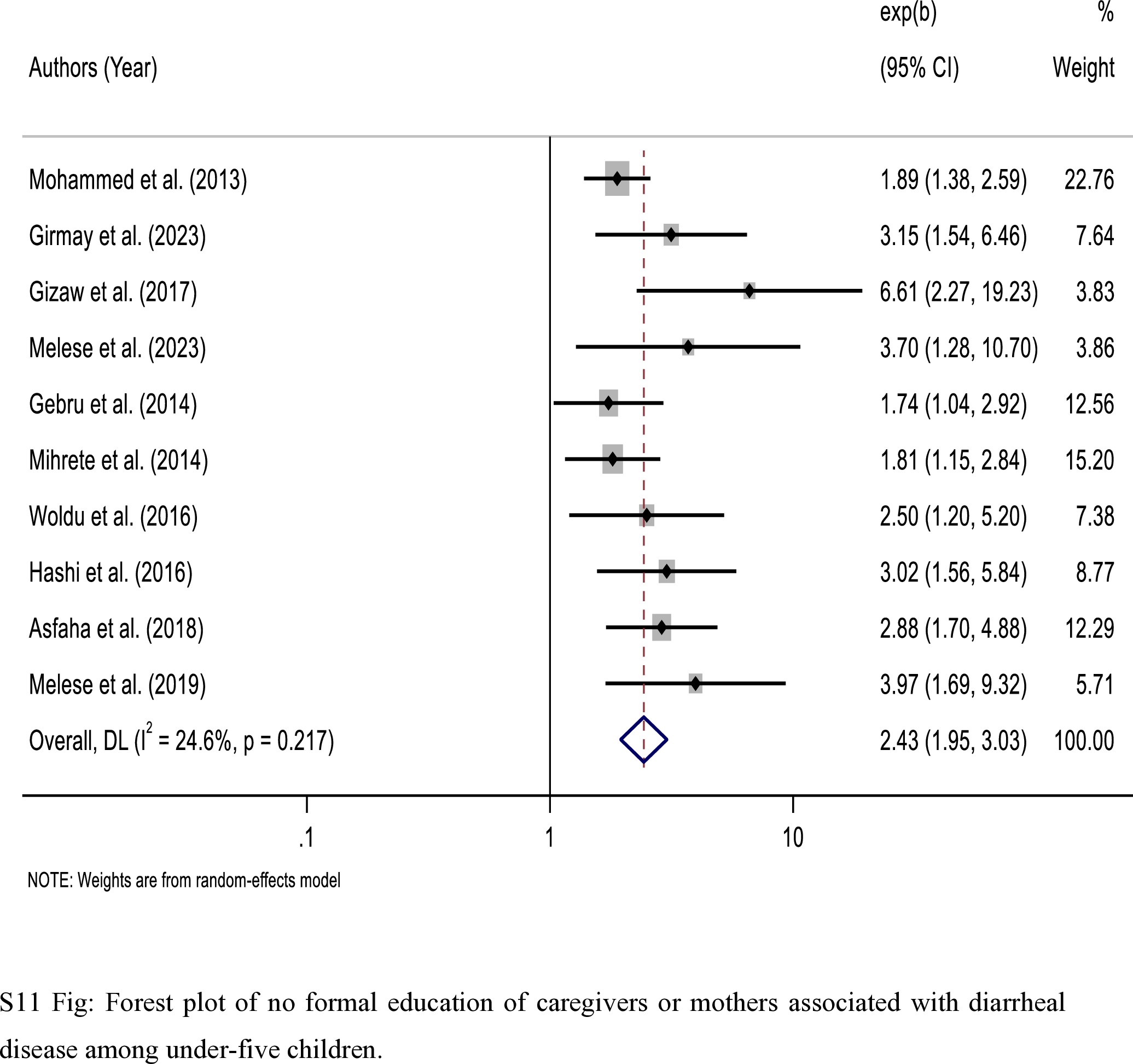

Supplement: S11 Fig — (TIF) [file pone.0326501.s015.TIF]

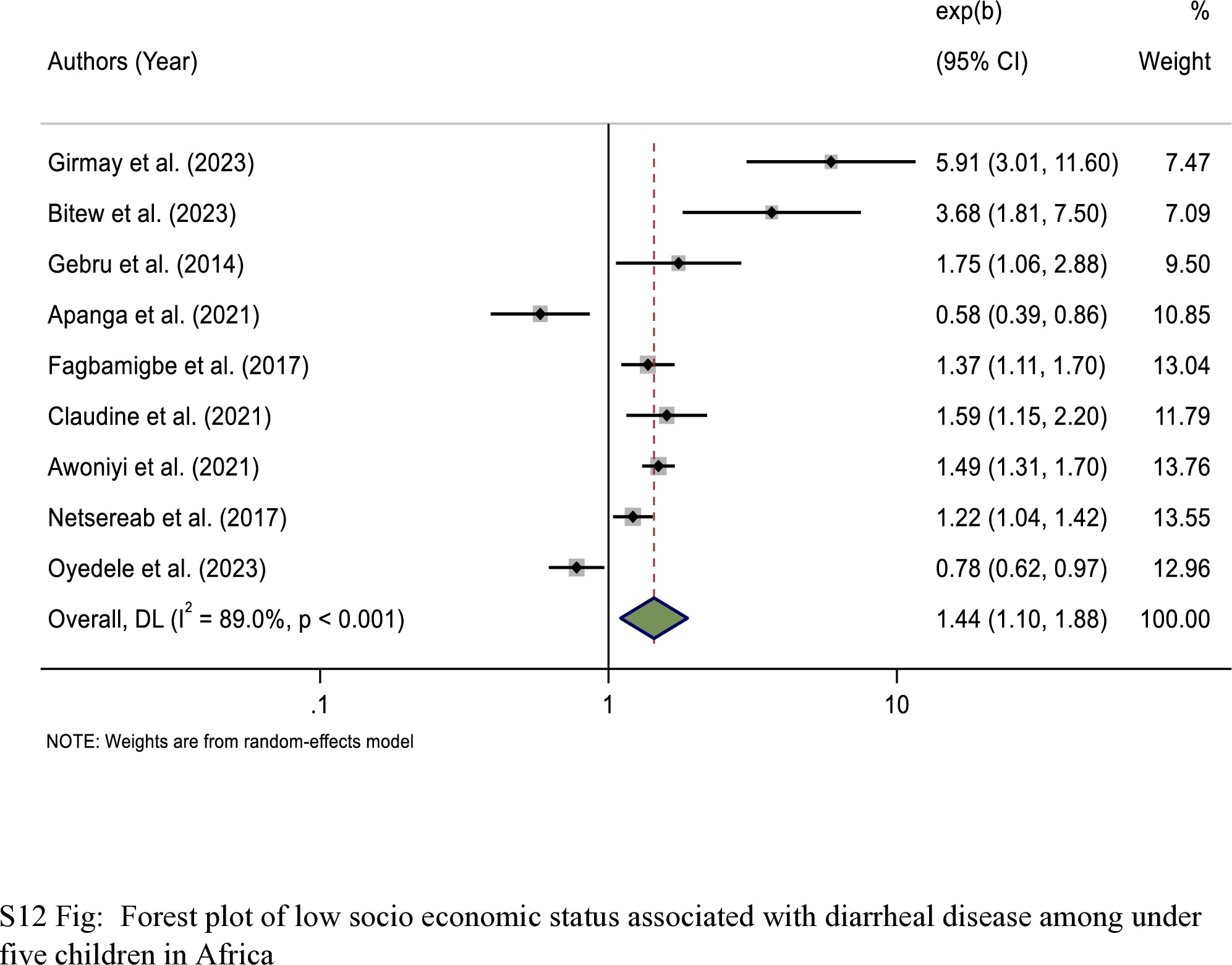

Supplement: S12 Fig — (TIF) [file pone.0326501.s016.TIF]

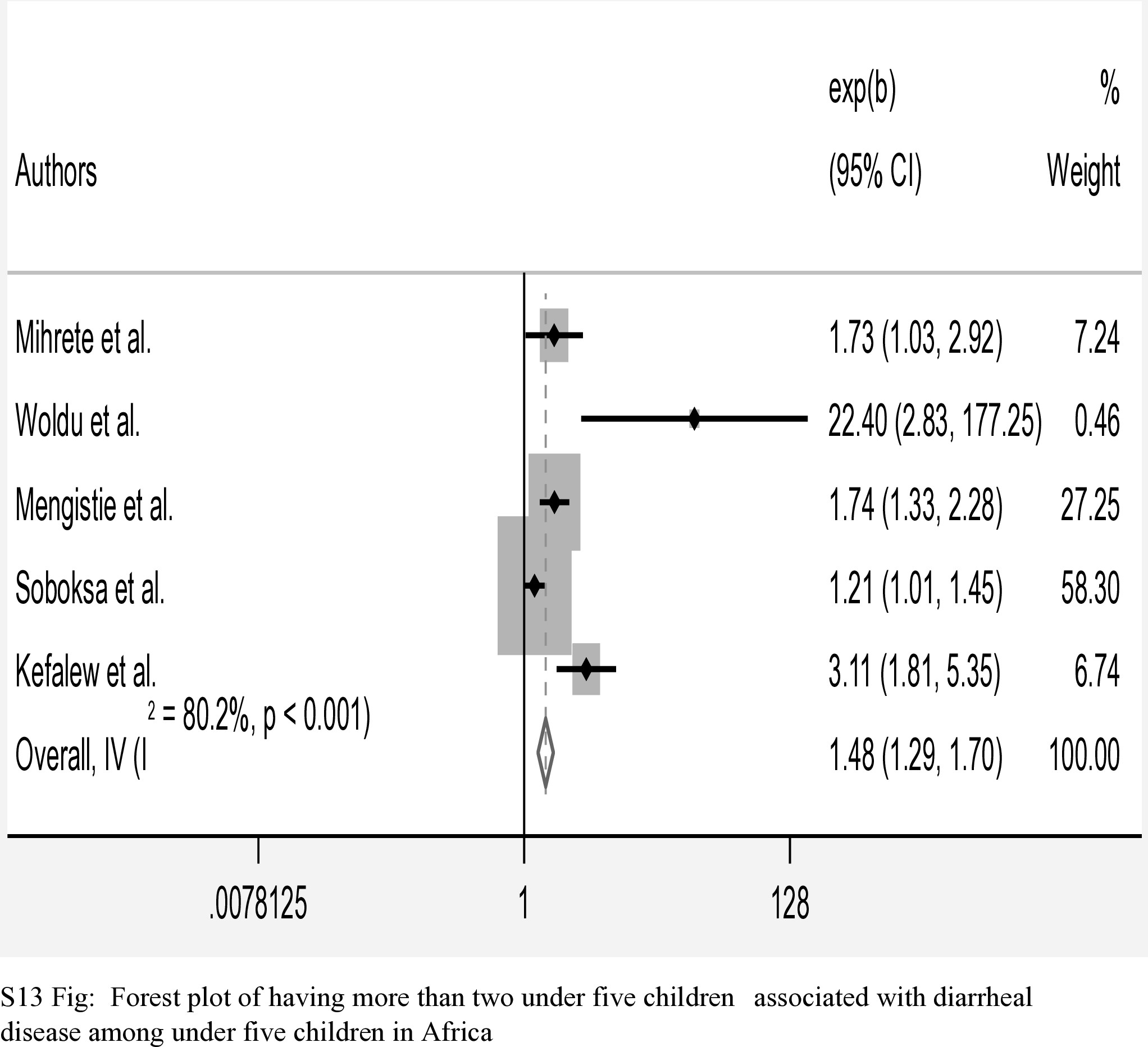

Supplement: S13 Fig — (TIF) [file pone.0326501.s017.TIF]

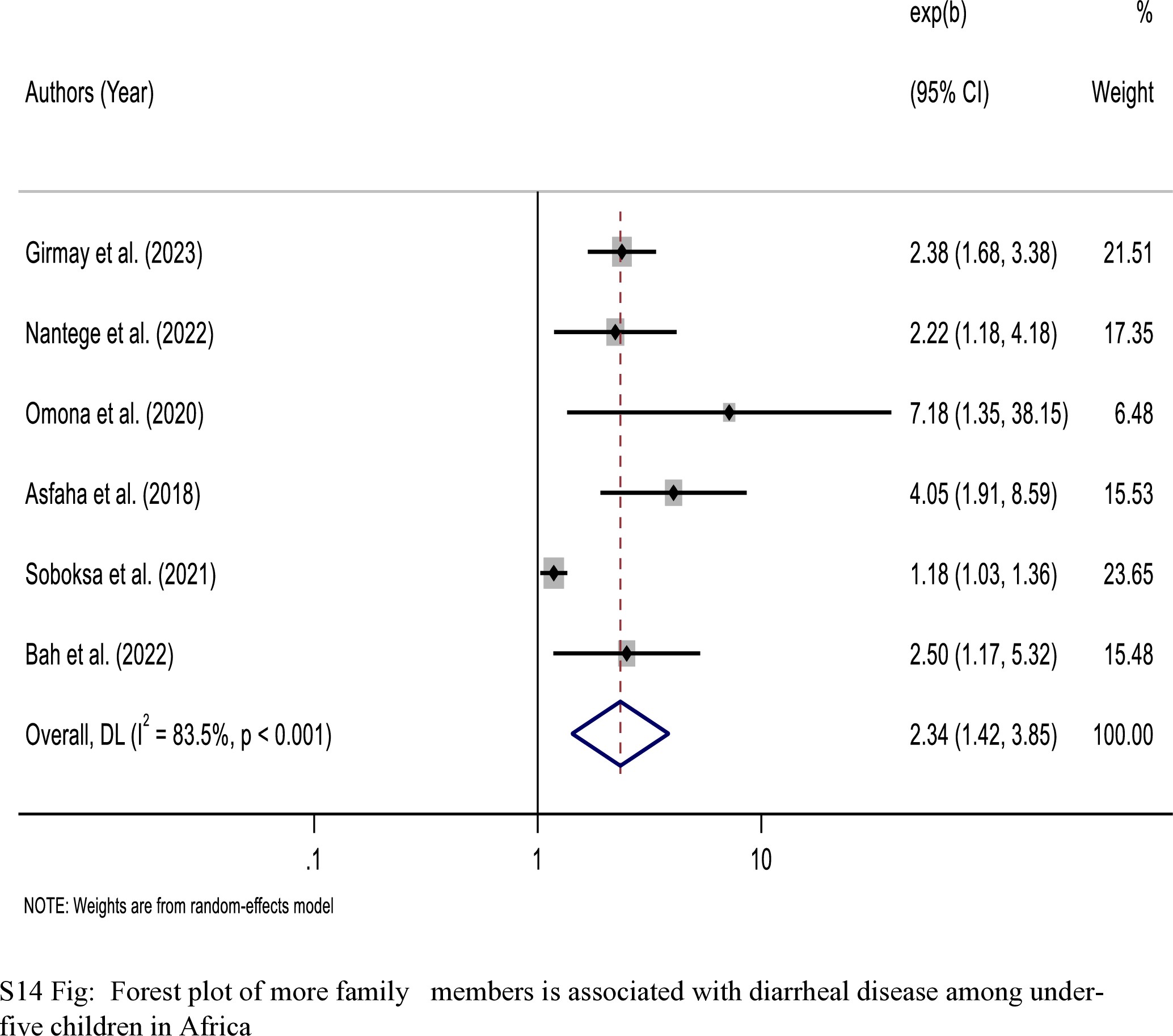

Supplement: S14 Fig — (TIF) [file pone.0326501.s018.TIF]
