# Supplementary material for: Prevalence of diarrheal diseases and associated factors among under five children in Africa: A meta-analysis
Source: PLoS One. 2025 Jul 3;20(7):e0326501. doi: 10.1371/journal.pone.0326501 (PMC12225826; doi:10.1371/journal.pone.0326501)
Supplement: Attachment — Submitted filename: REVIEWERS RECOMMENDATION- Diarrheal Disease.docx [file pone.0326501.s020.docx]

**REVIEWERS RECOMMENDATION**

The paper is a systematic review that focuses on diarrheal disease in children under the age of five in Africa from January 2013 to December 2023 using PRISMA guidelines. However, a lot must be done to make this ready for publication.

- The result section of the abstract needs to be rewritten for clarity. Improve sentence structure for readability.
- "Using a systematic search of PubMed, Scopus, Web of Science, and Google Scholar for studies from 2013–2023, following PRISMA guidelines" Is ambiguous. Kindly rewrite this part for clarity.
- There is no consistency in the font size usage and alignment of the paper. Some of the words are merged, such as **Sudan,64.2%(95%CI:59.71-68.68) (16), Uganda,62.4%(95%CI:57.55-67.25) (14) and Ethiopia ,41.75%(95%CI:40.53-42.97)(34)(Figure 2)** in Prevalence of Diarrheal disease among under-five children in Africa.
- The degree of freedom in Cochran's Q statistics for heterogeneity is missing for Central Africa.
- The dataset is dominated by studies from East Africa, with comparatively fewer studies from Central, Northern, and Southern Africa. This regional imbalance may skew the findings. Discussing how this affects generalizability would strengthen the conclusions.
  - The inclusion criterion limiting studies to those published in English may have excluded relevant research from French-speaking or Arabic-speaking African countries. This should be acknowledged.
- The heterogeneity (I² = 99.3%) is relatively high, which raises concern about the comparability of studies included in the review. Consider explaining how the high heterogeneity might impact the pooled prevalence estimates and associated risk factors.
- The plots are too compressed, especially the Sensitivity analysis and Funnel plots. Kindly revise all the plots to make them publication-worthy. The funnel plots suggested publication bias, which needs to be addressed.
- The Egger test was done with the number of studies to be 70, as indicated in the table, but your method alluded to only 69 studies.
- Here are some grammatical errors that needs to be addressed.
  - The title "Prevalence of diarrheal diseases and associated factors among under five children in Africa: Meta-analysis" should be corrected to "Prevalence of Diarrheal Diseases and Associated Factors Among Under-Five Children in Africa: A Meta-Analysis"
  - “A total of 69 studies meet inclusion criteria and included in the analysis” in the abstract should be corrected to “A total of 69 studies **met** inclusion criteria and **were** included in the analysis”.
  - In the introduction, “"Diarrheal diseases are continued public health problems in developing countries and the problem of diarrhea among under-five children is distributed unevenly across Africa” should be rewritten for clarity.
  - "The high mortality rates associated with diarrheal diseases are often linked to inadequate sanitation, poor hygiene practices, limited access to clean water, and under nutrition, which are common problems in many African communities (4, 5)." should be rewritten as “The high mortality rates associated with diarrheal diseases are often linked to inadequate sanitation, poor hygiene practices, limited access to clean water, and undernutrition, which are common problems in many African communities (4, 5)."
  - “In Africa, the burden of diarrheal diseases among children under five is further intensified by multiple socio-economic and health system challenges.” Should be rewritten as “In Africa, the burden of diarrheal diseases among children under five is further intensified by multiple socioeconomic and health system challenges”.
