# Supplementary material for: Prevalence of diarrheal diseases and associated factors among under five children in Africa: A meta-analysis
Source: PLoS One. 2025 Jul 3;20(7):e0326501. doi: 10.1371/journal.pone.0326501 (PMC12225826; doi:10.1371/journal.pone.0326501)
Supplement: Attachment — Submitted filename: Response to Reviewerss docx.docx [file pone.0326501.s022.docx]

**Dear Editor and Reviewers**

Please accept our revised manuscript and note our point-by-point response to the reviewer below for the manuscript titled ‹‹ **Prevalence of Diarrheal Diseases and Associated Factors among Under Five Children in Africa: A Meta-analysis**››.

Our revised manuscript continues to meet the journal’s formal requirements, including the abstract and overall word count.

We look forward to your reply and decision.

Thank you so much for your comments and constructive feedback.

Version: 2

Date: 5^th^ April/2025

Manuscript Number: PONE-D-24-52714R1.

**Author response to Reviewer #1:**

1. Please also indicate in the Methods section that you performed a meta-regression to quantify additional publication bias and conducted a sensitivity analysis to assess the robustness of your findings.

**Author reflection**: Comment accepted, and revision is done as A meta-regression analysis was also done to quantify additional publication bias. However, a sensitivity analysis was presented before this comment, which is «Sensitivity analyses were conducted to explore the robustness of the findings by excluding low-quality studies and studies with extreme prevalence estimates» (in the statistical analysis section, the last three lines).

2. In the Results section for subgroup analysis, after presenting the prevalence of each subgroup, clearly state whether there is a significant or non-significant difference among them. Based on the p-value provided, it appears there is a significant difference. Offer a concise interpretation of these differences. For example:

“Subgroup analysis by region revealed the highest prevalence in [Region A], followed by [Region B], [Region C], and [Region D]. There was a significant difference among these regions (p-value = …), indicating that each region exhibits distinct [example: characteristics/outcomes].”

**Authors Reflection:** Comment accepted and revision is done.

2. Please provide the DOI or URL for all references, along with the PubMed ID if available.

**Authors Reflection:** Dear reviewer thank you again for your constructive feedback. However, some papers not provid their DOI number and we try to present appropraite reference using Endote reference manager and hyperlink is done for all references.

3. The year for Danquah’s reference [67] is missing. Kindly re-check all references to ensure the accuracy of their publication years.

**Author Refelection**: Dear editors, thank you for your observation, Danquah’s publication year is 2014 and is checked.

4. On pages 14, 16, 17, 19, and 20, replace “Kefalew et al.” with “Alemayehu.”

**Authors’ reflection**: Dear Reviewers, thank you very much for your detailed observation, regarding your comments, there are two research papers in the author name start with Mulusew Alemayehu and Kefalew Alemayehu (Reference numbers 45 and 55 respectively). Therefore, we authors write this as Alemayehu M. et al and Kefalew A. et al) for better presentation and correction throughout the paper (Table 1 and 4).

5. On page 9, Nwokoro is cited as reference [57]; on page 10, Nwokoro is cited as reference [63]; and on page 11, Nwokoro is cited as reference [82]. Please verify that the reference numbering is consistent throughout the manuscript.

**Authors Refelection:** Yes, the comment is correct as it was in the revised truck changed manuscript, however, it was corrected in the clean manuscript in the previous reviewer's comments (because this research paper was written three times wrongly and was corrected in the previous comments) and currently only one Nwokoro author is present with the reference number of 57.

6. For references 69/61, confirm whether the author’s name is “Tampah-Naah” or “Naah.” Please ensure consistency in author names on pages 23–34 and 30–37.

**Author Reflection**: Thank you for your interesting comment. The correct author name is “Tampah-Naah,” and it has been used consistently throughout the revised manuscript. The reference is listed as [61] in the current clean version, and consistency has been ensured across all sections, including Tables 1 and 4.

7. On page 11, use a consistent font to report the meta-regression results.

**Author reflection:** Comment accepted and revision is done (font size is corrected 12 and New Times Roman,(Check the clean and truck change manuscript).

8. If feasible, consider including the PRISMA 2012 or PRISMA 2020 checklist as an additional file.

**Author Refelection**: Yes, the comment is acceptable, due to the attached additional file being more and we authors try to present this one in research paper screening process ( Figure1 PRISMA follow diagram).

9. Good luck!

Dear reviewers, we authors would like to thank you for your time and constructive feedback.

**Author Response to Reviewer #2 Comments**:

Our initial recommendations have mainly been resolved, but I found other minor issues that need to be addressed.

“Searches were conducted in databases including PubMed, Scopus, Web of Science, and Google Scholar to identified research ….” should be changed to “identify” in the method section.

**Authors Reflection:** Comment accepted and correction is done (check it in the abstract method section)

“exclusive breastfeeding (AOR = 3.06, 95%CI: 2.12-4.43)” There is still no consistency in writing the figures as seen in ventilated improved pit latrines (AOR = 0.85, 95% CI: 0.77-0.95). Please keep to a consistent format of writing the “95% CI”.

**Author’s reflection**: Comment accepted and revision is done (95%CI to 95% CI that is space is added consist for others)

“Regarding the study setting, 56 articles were done community-based…” should be either “56 articles were community-based” or “56 articles were done in community-based.

**Author’s reflection**: Comment accepted and revision is done as «56 articles were done in community-based».

The reference at 53 and 54 are the same. Is there any reason why they are separated?

**Author reflection:** Thank you for your observation. You are correct; the references at 53 and 54 were the same. This duplication was previously addressed and corrected in response to earlier reviewer comments, and the correct citation now appears as reference number 49: Akinyemi YC. Spatial pattern and determinants of diarrhea morbidity among under-five-aged children in Lagos State, Nigeria. Cities & Health. 2022;6(1):180–91. The duplicate has been removed accordingly.

“The pooled prevalence of diarrheal disease among under-five children in Africa was found 23.59%” should be corrected.

**Author Reflection:** dear Reviewers, thank you this pooled prevalence is correct, the result of the pooled prevalence of 66 research papers which s 25.59% and found in Figure 2. Because no research paper is added or removed in the current review process and no need for revision this pooled prevalence of DD.

In your discussion, you said, “Furthermore, a meta-analysis of five and six studies revealed that households with more than two under-five children and those with larger family sizes had a higher likelihood of developing DD compared to households with fewer children and smaller family sizes”. Why the choice of five and six studies? Can you add the reference to the studies used in that sentence?

**Author Reflection**: Thank you for your insightful comment. The selection of five and six studies was based on the number of included studies that specifically identified having more than two under-five children and larger family size as significant factors associated with DD. We authors have included those references in this section (discussion) ( you can see or check the truck chang manuscripts)
