# Supplementary material for: Prevalence of diarrheal diseases and associated factors among under five children in Africa: A meta-analysis
Source: PLoS One. 2025 Jul 3;20(7):e0326501. doi: 10.1371/journal.pone.0326501 (PMC12225826; doi:10.1371/journal.pone.0326501)
Supplement: Attachment — Submitted filename: Reviewer Comments.docx [file pone.0326501.s019.docx]

**Reviewer Comments**

| **Page** | **Paragraph/Section** | **Sentence** | **Revise Suggestion** |
| --- | --- | --- | --- |
| 3 & 17 | 2 | “Previous studies have consistently reported high prevalence rates of diarrheal diseases among under-five children in Africa (14-16).” | Please check and recheck whether this is a new paragraph. Check other paragraphs too. |
| 3  6  17 | 1  Quality assessment  1^st^ paragraph |      | Please paraphrase. The similarity rates from the turn-it in is high for these paragraphs. |
| 5 | Data extraction | Do you use stataMP-17 as well? | If yes, please state. |
| 10 | Example: Paragraph 3 | Example: Paragraph 3 – “were four times more likely to develop diarrheal disease compared to children below **sex** months” | Some sentences need English language editing. Please use English language editing software such as Gra*m*rly or any English editing service companies. |
| 10  18 | Paragraph 1  Paragraph 1 | “I2=99.3%”  Sudan,64.2%(95%CI:59.71-68.68)  . in support | Some sentences need punctuation editing service. Please recheck all sentences using editing software such as Grammarly or any English editing service companies. |
| All | Citations and References | Citation numbers and references | Please use cross-reference or hyperlink to link the citation numbers to the references. |
| 7  14 & 27  8 & 33  25  28  8, 26, and 27  13 & 14 | Incongruence Author Names  Citation  Citation & Figure 2  Incomplete reference (example: No year).  Missing reference  Missing reference  Table 1  Table 1 | Example:   Atari et al. is 2021 or 2023?  William’s citation is 2022 or 2023?  Example:  49. Akinyemi YC. Spatial pattern and determinants of diarrhoea morbidity among under-five aged children in Lagos State NCHJ-.  Kefalew et al. 2021  There’s missing reference for Nwokoro 2018.  In the references, there are two (double) Nwokoro, 2020.  Missing year for Danquah 2014 reference  Missing references for Naah et al 2019  Double citations:  Thiam 2017  CAPSLOCK:  ELMANSSURY et al. (2022)(80) | Please revise to be the same as the reference: Getahun, not Getahunet.  Please revise. It should be 2023.  Please revise. It should be 2023.  Please recheck all the references, according to The PlosOne template. For this reference, the year should be: 2019.  Please add reference for Kefalew 2021  Please add the reference for Nwokoro (2018).  Please add the year  Please add the reference  Please delete one of them  Please check any capslock or other size format for all text besides this citation |
| 33  9  Appendix（Sub group of prevalence） | Overall prevalence  Figure 2 on page 33 do not captured this data  Number of studies in Sub Group analysis | Incongruence overall prevalence between figure 2 and 3  (95% CI: 21.3 - 24.8; I2 = 99.3%). Based on this estimate, the lowest prevalence of diarrheal disease was found in Nigeria, 7.5% (95% CI: 5.76-9.24) (49), and the highest prevalence of diarrheal disease was found in South Africa, 67.3% (95% CI: 63.21–71.38) (15), South Sudan,64.2%(95%CI:59.71-68.68) (16), Uganda,62.4%(95%CI:57.55-67.25) (14) and Ethiopia ,41.75%(95%CI:40.53-42.97) (34)(Figure 2).  It would be beneficial to include a tree plot alongside Figure 2, presenting subgroup names and the number of studies in each group. | Please re-check, the overall score is not the same. Figure 2 23%, figure 3 16%. Is this because there’s double citation: Thiam 2017 in figure 2? There should be table presentation like this after table 1, for example: Area \| Pooled prevalence \| 95% CI; All Africa \| 7.5% \| 5.76 - 9.24; South Africa \| 67.3% \| 63.21 – 71.38 Etc. |
| 9 | Table 2 | The Df is empty | Please add the Df number |
| 11 | Incongruence number of reported studies and in the factors table data | In the results, there were **8 studies** mentioned. But in the Table, there are **10 studies** data for **poor hand washing factor** | Please recheck the number of studies in each subgroup, as well as the number of studies for other factors. |
| 14 & 15 | Factors order Table | Example: in the table, Poor solid and poor liquid management were separated by six factors | It had better to rearrange the order so that poor solid and poor liquid waste management is closed enough for readability. Please check others e.g. breast feeding, etc. |
| 16 | Discussion Paragraph 1 | At least 3-5 sentences in one paragraph. | Please discuss the Africa regional prevalence as well.  “The prevalence in… (Example South Africa) was….than in…. )  Discuss as well the contribution of the study findings for the research area, example:  This study updates the existing evidence on the prevalence of…… among …over the past decade, as well as identifying the various risk factors…. |
| 18 | Discussions paragraph 1&3 | Wordy | If possible, please make the sentences more concise. |
