# Supplementary material for: Prevalence of diarrheal diseases and associated factors among under five children in Africa: A meta-analysis
Source: PLoS One. 2025 Jul 3;20(7):e0326501. doi: 10.1371/journal.pone.0326501 (PMC12225826; doi:10.1371/journal.pone.0326501)
Supplement: Attachment — Submitted filename: Author Response for reviewer Comments Plos One (2).pdf [file pone.0326501.s021.pdf]

Dear Editor and Reviewers

Please accept our revised manuscript and note our point-by-point response to the reviewer below for the manuscript titled ‹‹ **Prevalence of Diarrheal Diseases and Associated Factors among under Five Children in Africa: A Meta-analysis**››.

Our revised manuscript continues to meet the journal's formal requirements, including the abstract and overall word count.

We look forward to your reply and decision.

Thank you so much for your comments and constructive feedback for better presentation of the manuscript. Our point-by-point responses are below.

Version:1

Date: 7th March/2025

Manuscript Number: PONE-D-24-52714

Reviewer Comments1#

**Page**	**Paragraph/Section**	**Sentence**	**Revise Suggestion**	**Author Response**
3 & 17	2	"Previous studies have consistently reported high prevalence rates of diarrheal diseases among under-five children in Africa (14-16)."	Please check and recheck whether this is a new paragraph. Check other paragraphs too.	Comment accepted and recheck is done and it is a new paragraph(for paragraph 3 in the background section.

3<br><br><br><br><br><br>6<br><br><br><br><br><br><br><br>17	1<br><br><br><br><br><br>Quality assessment<br><br><br><br><br><br><br><br>1st paragraph	According to the World Health Organization, data from 2021 indicated that diarrheal disease is responsible for 8% of deaths in children aged below 5 years (1). Diarrheal diseases are leading causes of preventable deaths, especially among children under five in developing countries (2). Diarrheal diseases are continued public health problems in developing countries and the problem of diarrhea among under-five children is distributed unevenly across Africa (3). The high mortality rates associated with diarrheal diseases are often linked to inadequate sanitation, poor hygiene practices, limited access to clean water, and under nutrition, which are common problems in many African communities (4, 5). These factors create a conducive environment for the transmission of diarrheal pathogens, exacerbating the health crisis faced by vulnerable populations (6, 7).<br><br>2.5. **Quality Assessment**<br><br>The quality of the included studies was assessed using the Joanna Briggs Institute (JBI) Critical Appraisal Checklist for prevalence studies (22). The quality assessment tool includes; (1) Were the criteria for inclusion in the sample clearly defined; (2) Were the study subjects and the setting described in detail; (3) Was the exposure measured in a valid and reliable way; (4) Were objective, standard criteria used for measurement of the condition; (5) Were confounding factors identified; (6) Were strategies to deal with confounding factors stated; (7) Were the outcomes measured in a valid and reliable way and (8) Was appropriate statistical analysis used. Studies were rated as low, moderate, or high quality based on factors mentioned above and only studies rated as moderate or high quality were included in the meta-analysis. Disagreements that arose during the full-text quality assessment were resolved through evidence-based discussion with the involvement of other review authors.<br><br>23 months were more likely to have diarrheal disease compared to lower age categories. This may be due to the fact that children aged more than six months start crawling or walking which increases their exposure to infectious agents (37). Furthermore, when children aged six months start complementary feeding this may increase their exposure to different types of infections through contaminated food and water (43, 52). In addition, children who were not exclusively	Please paraphrase. The similarity rates from the turn-it-in are high for these paragraphs.	The comment accepted and paraphrasing is done. In addition, acknowledgment through citation is used.<br><br>Regarding the Quality assessment checklist: revision is done using the Newcastle quality assessment tool. All the comments raised by the reviewer are checked and the similarity is solved in the new version.<br><br>Comment accepted and revision is done (cross check the manuscript with revised truck change manuscript).
5	Data extraction	Do you use stataMP-17 as well?	If yes, please state.	For data extraction, we used an Excel spreadsheet, but for analysis, we used Stata version 17 software.
10	Example: Paragraph 3	Example: Paragraph 3 – "were four times more likely to develop diarrheal disease compared to children below **sex** months"	Some sentences need English language editing. Please use English language editing software such as Gra*m*rly or any English editing service company.	Comment accepted and the revision is done. We used grammarly software for different services including punctuation editing.
10	Paragraph 1	"I2=99.3%"<br>Sudan,64.2%(95%CI:59.71-68.68)	Some sentences need a punctuation editing service. Please recheck all sentences using editing software such	Comment accepted and we used Grammarly to correct different grammar errors including punctuations.

18	Paragraph 1	. in support	as Grammarly or any English editing service company.	
All	Citations and References	Citation numbers and references	Please use cross-reference or hyperlink to link the citation numbers to the references.	Comment Accepted and the hyperlink or cross-reference citation is added.
7<br><br>14 & 27<br><br>8 & 33<br><br>25<br><br>28<br><br>8, 26, and 27<br><br>13 & 14	Incongruence Author Names<br><br>Citation<br><br>Citation & Figure 2<br><br>Incomplete reference (example: No year).<br><br>Missing reference<br><br>Missing reference	Example: Author, year of publication and Reference / Tadesse et al. (2022)(23) / Getahunet al. (2021)(24)<br><br>Atari et al. is 2021 or 2023?<br><br>William's citation is 2022 or 2023?<br><br>Example:<br>49. Akinyemi YC. Spatial pattern and determinants of diarrhoea morbidity among under-five aged children in Lagos State NCHJ-.<br><br>Kefalew et al. 2021<br><br>There's missing reference for Nwokoro 2018.<br>In the references, there are two (double) Nwokoro, 2020.	Please revise it to be the same as the reference: Getahun, not Getahunet.<br><br>Please revise. It should be 2023.<br><br>Please revise. It should be 2023.<br><br>Please recheck all the references, according to The PlosOne template. For this reference, the year should be: 2019.<br><br>Please add reference for Kefalew 2021<br><br>Please add the reference for Nwokoro (2018).<br><br>Please add the year	Comment accepted and revision is done, yes some references is directly taken from stata command is generated like getahunnet. But we authors correct it accordingly the comment is given.<br>Its corrected as 2023.<br><br>Comment Accepted and revised as William et al 2023 , both in Table 1 and figure 2.<br><br>Comment accepted and all reference is cheked and corrected<br><br>Kefalew et al I have checked and it is correct.<br><br>Dear editors, thank you for detail comments in the case of Nwokoro 2020, one reserch paper was enter twice, so we authors corrected it by removing one of them and 2018 is not correct ,it was 2020. All are was about Nwokoro 2020. So one is used for data analysis(Nwokkoro 2018 is removed).<br><br>Comment accepted and the year is added(Danquah 2014)<br><br>The comment has been accepted and the reference is added.<br><br>The comment is accepted, and one is deleted

<table>
<tr>
<td></td>
<td>Table 1

Table 1</td>
<td>Missing year for Danquah 2014 reference

Missing references for Naah et al 2019

Double citations:
Thiam 2017

CAPSLOCK:
ELMANSSURY et al. (2022)(80)</td>
<td>Please add the reference

Please delete one of them

Please check any capslock or other size format for all text besides this citation.</td>
<td>The comment is accepted and the reference is added(Naah et al 2019)

Comment is accepted and one is deleted(Thiam, 2017)
The comment is accepted, and caps lock is corrected (Elmanssury et al. 2022)</td>
</tr>
<tr>
<td>33

9</td>
<td>Overall prevalence

Figure 2 on page 33 do not captured this data</td>
<td>Incongruence overall prevalence between figure 2 and 3

(95% CI: 21.3 - 24.8; I2 = 99.3%). Based on this estimate, the lowest prevalence of diarrheal disease was found in Nigeria, 7.5% (95% CI: 5.76-9.24) (49), and the highest prevalence of diarrheal disease was found in South Africa, 67.3% (95% CI: 63.21–71.38) (15), South Sudan,64.2%(95%CI:59.71-68.68) (16), Uganda,62.4%(95%CI:57.55-67.25) (14) and Ethiopia,41.75%(95%CI:40.53-42.97) (34)(Figure 2).</td>
<td>Please re-check, the overall score is not the same. Figure 2 23%, figure 3 16%. Is this because there’s double citation: Thiam 2017 in figure 2?

There should be table presentation like this after table 1, for example:

<table><tr><td>Area</td><td>Pooled prevalence</td><td>95% CI</td></tr><tr><td>All Africa</td><td>7.5%</td><td>5.76 - 9.24</td></tr><tr><td>South Africa</td><td>67.3%</td><td>63.21 – 71.38</td></tr></table>
Etc.</td>
<td>Comment accepted and revision is done. The overall prevalence in Africa and regional sub group pooled prevalence is estimated using a forest plot(Figure 2).

Dear Reviewers, this data is directly taken from Table 1 as it is extracted from the source no additional analysis was done for this purpose.

Constructing this recommended table is best, but it already includes the pooled prevalence of DD in all of Africa, and each region is presented in forest plot in Figure 2. (If needed, I will do it in the next comment for additional revision).

The comment is accepted, and the tree plot alongside Figure 2 is done, including subgroup analysis by region as East Africa(EA), West Africa(WA), North Africa(NA), South Africa (SA), and Central Africa (CA).</td>
</tr>
</table>

Appendix（Sub group of prevalence）	Number of studies in Sub Group analysis	It would be beneficial to include a tree plot alongside Figure 2, presenting subgroup names and the number of studies in each group.		
9	Table 2	The Df is empty	Please add the Df number	Comment accepted and degree freedom is corrected(2)
11	Incongruence number of reported studies and in the factors table data	In the results, there were **8 studies** mentioned. But in the Table, there are **10 studies** data for **poor hand washing factor.**	Please recheck the number of studies in each subgroup, as well as the number of studies for other factors.	Comment accepted and correction is done as it found in the table, there is nine studies (there was one study was double (Nantege et al). So the correct one is nine studies and the correction is done with nine studies(In table 4).
14 & 15	Factors order Table	Example: in the table, Poor solid and poor liquid management were separated by six factors	It had better to rearrange the order so that poor solid and poor liquid waste management is closed enough for readability. Please check others e.g. breast feeding, etc.	The comment was accepted, and the revision is done.
16	Discussion Paragraph 1	At least 3-5 sentences in one paragraph.	Please discuss the Africa regional prevalence as well.<br>"The prevalence in… (Example South Africa) was….than in….)<br><br>Discuss as well the contribution of the study findings for the research area, example:<br>This study updates the existing evidence on the prevalence of……	The comment is accepted, and the revision is done(paragraph 1,2 and 3.)

			among …over the past decade, as well as identifying the various risk factors….	
18	Discussions paragraph 1&3	Wordy	If possible, please make the sentences more concise.	Comment accepted and revision is done.
**Reviewer 2# Comments and suggestions**				
1	Paragraph section	The paper is a systematic review that focuses on diarrheal disease in children under the age of five in Africa from January 2013 to December 2023 using PRISMA guidelines. However, a lot must be done to make this ready for publication		Comment accepted and revision is done(cross check clean with truck changed manuscript).
2	Abstract result section	The result section of the abstract needs to be rewritten for clarity. Improve sentence structure for readability.		Comment accepted and revision is done
		Using a systematic search of PubMed, Scopus, Web of Science, and Google Scholar for studies from 2013–2023, following PRISMA guidelines" Is ambiguous. Kindly rewrite this part for clarity.		Comment is accepted and it is revised as: Search databases including PubMed, Scopus, Web of Science, and Google Scholar were used to find the research from January 2013 to December 2023. The PRISMA flow chart guideline was used to screen the research.
	Result section	There is no consistency in the font size usage and alignment of the paper. Some of the words are merged, such as Sudan,64.2%(95%CI:59.71-68.68) (16), Uganda,62.4%(95%CI:57.55-67.25) (14) and Ethiopia ,41.75%(95%CI:40.53-42.97)(34)(Figure 2) in Prevalence of Diarrheal disease among under-five children in Africa.		Comment accepted and revision is done.
		The degree of freedom in Cochran's Q statistics for heterogeneity is missing for Central Africa.		Comment accepted and corrected df which was 2 .
		The dataset is dominated by studies from East Africa, with comparatively fewer studies from Central, Northern, and Southern Africa. This regional imbalance may skew the findings. Discussing how this affects generalizability would strengthen the conclusions.		Comment accepted, considering this we author try to conduct sub group analysis by region and indicate pooled prevalence for each region.

		The inclusion criterion limiting studies to those published in English may have excluded relevant research from French-speaking or Arabic-speaking African countries. This should be acknowledged.	Comment accepted and acknowledged in limitation section.
		The heterogeneity ($I^2 = 99.3\%$) is relatively high, which raises concern about the comparability of studies included in the review. Consider explaining how the high heterogeneity might impact the pooled prevalence estimates and associated risk factors.	Dear reviewer, considering this, sub group analysis were done and meta regression also done sample size, year of publication and the region with standard error take as covariate.
		The plots are too compressed, especially the Sensitivity analysis and Funnel plots. Kindly revise all the plots to make them publication-worthy. The funnel plots suggested publication bias, which needs to be addressed.	Dear reviewers due to included studies were large (66) sensitivity and Funnel plot is compressed. We authors try to modify the funnel plot and sensitivity analysis. Then, the revised one is present better even the included study is large (66) for this purpose.
